# Paraphenylenediamine (Hair Dye) Poisoning: A Prospective Study on the Clinical Outcome and Side Effects Profile

**DOI:** 10.7759/cureus.29133

**Published:** 2022-09-13

**Authors:** Sohaib Asghar, Summayha Mahbub, Shoaib Asghar, Salman Shahid

**Affiliations:** 1 Emergency Medicine, Glan Clwyd Hospital, Rhyl, GBR; 2 Internal Medicine, Glan Clwyd Hospital, Rhyl, GBR; 3 Medicine, Sheikh Zayed Medical College/Hospital, Rahim Yar Khan, PAK

**Keywords:** acute kidney injury, rhabdomyolysis, angioedema, suicide, creatine phosphokinase (cpk), poisoning, paraphenylene-diamine (ppd)

## Abstract

Background: Systemic poisoning with paraphenylenediamine (PPD) also known as Kalapathar, is an emerging suicidal trend in developing south Asian and African countries. The clinical distinction of hair dye toxicity comprises severe angioedema of the face and neck, tongue swelling resulting in upper airway obstruction, acute liver injury, myocarditis, and rhabdomyolysis complicating to acute kidney injury.

Aim: To raise awareness, document the characteristic clinical spectra, and prevent and predict the outcome of poisoning (suicidal or accidental) with PPD (hair-dye) based on clinical complications, early baseline laboratory results and creatine phosphokinase (CPK) levels monitoring.

Place and duration: Department of Medicine and Emergency, Sheikh Zayed Hospital, Rahim Yar Khan. One year of study from August 19, 2021 to August 17, 2022.

Methods: A total of 103 patients, with no comorbidities, who presented with acute PPD poisoning were included in this study. The demographic profile, clinical features, laboratory results, route and mode of intoxication were noted in a special proforma. Furthermore, clinical outcomes in the form of need for tracheostomy or mechanical ventilation, mortality and mean hospital stay were also documented. The percentages were calculated for categorical data like demographic profile, clinical features and clinical outcomes. Mean and standard deviation was calculated for continuous variables, i.e., laboratory parameters.

Results: Out of 103 patients, 88 (85.4%) were females who belonged to a low socioeconomic class (89%). The mean age of the patients was 26.39 ± 3.41 years. The majority of cases were suicidal self-poisoning (98%), and the route was oral (98%). In 82 (79.6%) of the patients, the characteristic cervicofacial angioedema, dysphagia, dysphonia, tongue swelling and stridor were noted. Clinical complications such as rhabdomyolysis (67.9%), chocolate-colored urine (82.5%), hepatitis (58.2%), and acute kidney injury (22.3%) were noted in the later clinical stages of PPD poisoning. Emergency tracheostomy was performed in 77.6% of patients. The mortality rate in this study stands at 12.6% and the mean hospital stay at 6.25 ±3.99 days. The mean and standard deviation of serum creatinine, CPK, alanine aminotransferase (ALT), aspartate aminotransferase (AST), total leukocyte count (TLC), and serum potassium were, respectively, noted at 2.431 ± 2.275 mg/dL, 1090.8 ± 218.6 IU/L, 476.8 ± 1038.8 IU/L, 639.1 ± 1006, 11100 ± 4124.1 cells/mm3, and 4.8 ± 1.061 mmol/L.

Conclusion: PPD is emerging as the poison of choice in suicidal young female patients due to its easy, low-cost availability and higher mortality. The cervicofacial angioedema, tongue swelling and rhabdomyolysis impending acute kidney injury are hallmarks of PPD poisoning. The treatment is largely supportive with no specific antidote available. Early clinical diagnosis and supportive therapeutic management in the form of maintenance of airway patency, timely tracheostomy with post-operative tube care, intravenous medications (fluids, steroids, antihistamine), and renal dialysis can save lives and may lead to full recovery. In addition, strict legal measures should be endorsed to ban the sale and use of lethal PPD in hair dyes.

## Introduction

Suicide is a preventable public health problem, where more than 700,000 people die every year and for each suicidal death, there are more than 20 suicide attempts [1]. Toxicity with hair dye containing paraphenylenediamine (PPD) is an emerging trend [1] of suicidal poisoning, especially in developing African and Asian countries, and is associated with higher morbidity and mortality [2].

PPD is abundantly found in “Kalapathar (black stone).” It is usually added in henna (*Lawsonia alba*) after crushing, which is then applied as a form of popular custom to stain the soles and palms, and as a red or black hair dye. PPD lessens the amount of henna needed, intensifies its color, and hastens the staining process. Local application of PPD may cause local irritation, urticaria, arthritis, asthma, lacrimation or blindness if applied to the eyes [3,4]. Oral consumption of 7-10g as a lethal dose of PPD in our patients results in sudden tongue swelling, dysphagia, dysphonia, stridor and severe cervicofacial edema often requiring emergency tracheostomy to relieve respiratory obstruction [5-7]. This often leads to acute hepatitis (15%-68%), myocarditis (12%-20%), rhabdomyolysis (20%-69%), myoglobinuria (30%-84%), and acute kidney injury with a prevalence from 20% to 84% [5,6]. Complications are dose-dependent and are managed conservatively [8]. Despite high morbidity and mortality even in moderate doses, no specific antidote is available [8].

The study aimed to raise awareness, document the characteristic clinical spectra, and prevent and predict the outcome of PPD poisoning based on clinical complications, early baseline laboratory results and creatine phosphokinase (CPK) levels monitoring.

## Materials and methods

Operational definition

PPD Intoxication

Diagnosis is based on the history of ingestion from the patient or relative, characteristic clinical manifestations (cervicofacial edema with or without shortness of breath and/or black color urine) and raised CPK levels (normal range of CPK = 10-120 microgram per liter [mcg/L]).

Angioedema

Angioedema is an area of swelling (edema) of the lower layer of skin and tissue just under the skin or mucous membranes. The swelling may occur on the face, tongue and neck.

Acute Hepatitis

Increase in serum ALT >80 IU/L

Rhabdomyolysis

An increase in serum CPK levels >2-3 times the reference range of 50-250 IU/L with a suspected history of poisoning within 12 hours is suggestive of early rhabdomyolysis, though CPK levels in rhabdomyolysis are as high as 100 times.

Acute Kidney Injury

Increase in serum creatinine by 0.3mg/dL or more within 48 hours, or greater than 1.5 times baseline creatinine or urine output less than 0.5mL per kg per hour for six hours.

Low Socioeconomic Status

This refers to individuals having less access to financial, educational, social, and health resources than those with a higher socioeconomic status.

Study design

The prospective cohort study was carried out in the Department of Medicine and Emergency, Sheikh Zayed Hospital, Rahim Yar Khan. The study was conducted from August 19, 2021 to August 17, 2022. The inclusion criterion included the diagnosis of PPD poisoning based on a patient history of oral ingestion or skin route intoxication and clinical manifestations. Patients with any medical conditions, drug history, co-morbidities (renal, hepatic and cardiac), or previous exposure were excluded from the study.

Data collection procedure

After approval of the research study from the Institutional Ethical Review Board, a total of 103 patients from the department of Medicine and Emergency, Sheikh Zayed Hospital, Rahim Yar Khan fulfilling the inclusion criteria were selected. The purpose of the study was explained to each patient and informed consent was taken. The demographic profile such as age, gender, marital status, socioeconomic status, route and mode of intoxication were noted in a proforma.

Baseline investigations like complete blood count (CBC), liver function tests (serum ALT and AST), renal function test (serum creatinine), serum potassium and CPK levels were sent to a laboratory for all selected patients. Renal imaging was done in all patients to rule out any obstructive or previous pathology, whereas urine examination for renal tubular casts was excluded from proforma as performed only on patients with rhabdomyolysis or acute kidney injury. Findings were entered in pre-designed proforma questionnaire. Furthermore, clinical outcomes in the form of need for tracheostomy, mechanical ventilation, renal dialysis, hospitalization time and mortality rate were also recorded.

Data analysis

Data analysis was computer-based by statistical software SPSS version 22 (IBM Corp., Armonk, NY). The percentages were calculated for categorical data like age, gender, marital status, socioeconomic status, clinical features, route of intoxication (oral or dermal), and mode (suicidal or accidental) of intoxication. Mean and standard deviation was calculated for continuous variables, i.e., laboratory parameters like serum creatinine, CPK levels, AST/ALT levels, TLC count and serum potassium.

## Results

A total of 103 patients with PPD poisoning were selected for this study. Among these patients, 88 (85.4%) were females, while 15 (14.6%) were males. The mean age of the patients was 26.39 ± 3.41 years. The majority of cases were suicidal self-poisoning (98%), the route was oral (98%), and belonged to the low socioeconomic class (89%) (Table 1).

**Table 1:** Demographic features of paraphenylenediamine (hair dye) poisoning patients

Demographic features	Number of cases	Percentages (%)
Age 15-20 (Years)	4	3.9
Age 21-30 (Years)	93	90.3
Age 31-40 (Years)	6	5.8
Gender - Female	88	85.4
Gender - Male	15	14.6
Socioeconomic Status - Lower	92	89
Socioeconomic Status - Middle	11	11
Mode of Intoxication - Suicidal	100	98
Mode of Intoxication - Accidental	03	02
Route of Intoxication - Oral	100	98
Route of Intoxication - Dermal	03	02

Eighty-two patients (79.6%) had the characteristics of cervicofacial angioedema, dysphagia, dysphonia, tongue swelling and stridor in the first 2-6 hours of a lethal dose of 7-10g ingestion. Clinical outcomes such as rhabdomyolysis (67.9%), chocolate-colored urine (82.5%), acute hepatitis (58.2%), and acute kidney injury (22.3%) were noted in the later (6-72 hours) clinical stages of PPD poisoning (Table 2).

**Table 2:** Clinical profile of paraphenylenediamine (hair dye) poisoning patients

Clinical features	Number ofcCases	Percentages (%)
Severe Cervicofacial edema	82	79.6
Tongue Swelling	82	79.6
Dysphagia	82	79.6
Dysphonia	82	79.6
Stridor	82	79.6
Rhabdomyolysis	70	67.9
Chocolate colored Urine	85	82.5
Acute Hepatitis	60	58.2
Acute Kidney Injury	23	22.3

Emergency tracheostomy was done in 77.6% of patients whereas 1.9% needed mechanical ventilation. Out of 23 patients (22.3%) who developed acute kidney injury in 6-72 hours of lethal PPD dose, only five patients (4.8%) had undergone renal replacement therapy. The mean hospital stay, and mortality were 6.25 ±3.99 days and 12.6% (Table 3).

**Table 3:** Clinical outcome of paraphenylenediamine (hair dye) poisoning patients

Clinical outcome	Number of cases	Percentages (%)
Tracheostomy	80	77.6
Mechanical Ventilation	02	1.9
Renal Replacement Therapy	05	4.8
Mean Hospital Stay	6.25 ± 3.99 days	
Mortality Rate	13	12.6

The mean and SD of serum creatinine, CPK, ALT, AST, TLC, and serum potassium were, respectively, noted and displayed in Table 4.

**Table 4:** Laboratory parameters of paraphenylenediamine (hair dye) poisoning patients

Laboratory parameters	Mean	± SD	Mode	Range
Serum Creatinine, mg/dL	2.431	2.275	0.9	0.5–07
Creatine Phosphokinase (CPK), IU/L	1090.8	218.6	198	198-12045
Alanine Aminotransferase (ALT), IU/L	476.8	1038.8	7–56	34-3326
Aspartate Aminotransferase (AST), IU/L	639.1	1006	10-40	118-3,210
Total Leukocyte Count (TLC), cells/mm^3^	11,100	4,124.1	5,600	5,000-15,300
Serum Potassium, mmol/L	4.8	1.061	3.6	3.6–6

## Discussion

Kalapathar (black stone) containing PPD, an easily available and cheap hair dye, is rising as a common suicidal substance in the developing world involving those primarily of low socioeconomic status and rural inhabitants [2,4]. It contains toxins including PPD, sodium ethylene diamine tetra acetic acid, and propylene glycol which can result in multiorgan failure [5-9].

In our analysis, the prevalence of self-maltreatment by ingesting kalapathar was more in the age group of 21-30 years with an 85.4% majority as female. A parallel age range with female preponderance was noted by Akber et al. [2], Qasim et al. [9], and Chrispal et al. [10]. This fact of female preponderance in PPD poisoning could be clarified by easy accessibility and the low cost of hair dye [2,11,12]. In addition to that, females are more exposed to gender inequalities and social pressures in developing countries [9]. The preferred route in our study was oral with suicidal intent, the same as noticed by Kondle et al. [8], Qasim et al. [9], and Khan et al. [13]. The patients developed a varying degree of cervicofacial edema two to six hours after ingestion. The number of patients who developed severe angioedema, tongue swelling, stridor, and acute respiratory obstruction was 82%, whereas emergency tracheostomy was performed in these 80 (77.6%) patients who then needed post-tracheostomy tube care with nebulization, suctioning and oxygen inhalation through this tube route. Two patients were put on mechanical ventilation due to severe cervicofacial edema, late presentation with respiratory failure and not maintaining saturation despite post-tracheostomy care. Tracheostomy insertion of 60% has been recorded in a study at Multan [2], Bahawalpur [9] 100%, Nawabshah [11] 87.5%, South India [14] 68.5% but 77.6% patients of in this study needed an emergency lifesaving tracheostomy to relieve upper airway tract obstruction.

Apart from respiratory complications, renal [5,6], hepatic [15], hematological [9,11-13] and cardiac manifestations [12-16] were also noted in acute poisoning with prevalence of 47%-90% [17-22]. In this study, myocarditis developed in 11% of patients, which is in line with a study done by Tanweer et al. [15] where myocarditis was reported at 27% and Khan et al. [16] noted myocarditis in 12% of patients. The acute hepatitis of 58.2% was noted in this study, as compared to 66.67% reported in a study conducted by Ahmad et al. [18].

The CPK levels [19] increase two to three times the reference range within 12 hours of ingestion suggesting early rhabdomyolysis (67.9%). The dark color urine (82.5%) with declining urine output and inclining serum creatinine with a mean and standard deviation of 2.431 ± 2.275, was suggestive of acute kidney injury which was 22.3% in this study. Renal replacement therapy in the form of hemodialysis was done in 4.8% of our patients. This was consistent with the studies of Gabow [5] and Averbukh [6] as well as Tanweer et al. [15] where rhabdomyolysis was evident in 91/122 (74.5%) patients, acute kidney injury [21-23] in 30% out of which 16 patients required renal replacement therapy (RRT) [15].

The mortality rate was 12.6% in our study, compared to Akbar et al, who reported it to be 20%, and Khuhro et al. [11] reported mortality to be 37.5% among their patients at Nawabshah hospital. The mortality of 22.48% was noted by Jain et al. [12] in a study conducted on 1,595 patients with PPD poisoning in Jhansi-India, whereas Nirmala et al. [14] reported 22.2% mortality. Another study at Dera Ismail Khan by Khan et al. [13] reported a mortality rate of 47.4% in patients with PPD poisoning. The mean hospital stay in this study was 6.25 ±3.99 days.

Limitations of the study were patients with any medical conditions, drug history, co-morbidities (renal, hepatic and cardiac), or previous exposure were excluded from the study.

## Conclusions

PPD is emerging as the poison of choice in suicidal young female patients due to its easy, low-cost availability and higher mortality. The cervicofacial angioedema, tongue swelling and rhabdomyolysis impending acute kidney injury are hallmarks of PPD poisoning. The treatment is largely supportive with no specific antidote available. Early clinical diagnosis and supportive therapeutic management in the form of maintenance of airway patency, timely tracheostomy with post-operative tube care, intravenous medications (fluids, steroids, antihistamine), and renal dialysis can save lives and may lead to full recovery. In addition, strict legal measures should be endorsed to ban the sale and use of lethal PPD in hair dyes.
